# Adherence to pan‐genotypic glecaprevir/pibrentasvir and efficacy in HCV‐infected patients: A pooled analysis of clinical trials

**DOI:** 10.1111/liv.14266

**Published:** 2019-10-18

**Authors:** Ashley Brown, Tania M. Welzel, Brian Conway, Francesco Negro, Norbert Bräu, Jason Grebely, Massimo Puoti, Alessio Aghemo, Henning Kleine, David Pugatch, Federico J. Mensa, Yaozhu J. Chen, Yang Lei, Eric Lawitz, Tarik Asselah

**Affiliations:** ^1^ Imperial College Healthcare NHS Trust London UK; ^2^ Department of Medicine 1 J. W. Goethe University Hospital Frankfurt am Main Germany; ^3^ Vancouver Infectious Diseases Center Vancouver BC Canada; ^4^ Divisions of Gastroenterology and Hepatology and Clinical Pathology Geneva University Hospital Geneva Switzerland; ^5^ James J. Peters VA Medical Center Icahn School of Medicine at Mount Sinai New York City NY USA; ^6^ The Kirby Institute UNSW Sydney Sydney NSW Australia; ^7^ Department of Infectious Diseases AO Ospedale Niguarda Ca' Granda Milan Italy; ^8^ Humanitas University and Clinical and Research Hospital Rozzano Italy; ^9^ AbbVie Deutschland GmbH & Co KG Wiesbaden Germany; ^10^ AbbVie North Chicago IL USA; ^11^ Texas Liver Institute University of Texas Health San Antonio San Antonio TX USA; ^12^ Department of Hepatology Centre de Recherche sur l'Inflammation (CRI) INSERM UMR 1149 University of Paris Diderot AP‐HP Hôpital Beaujon Clichy France

**Keywords:** adherence, G/P, glecaprevir, hepatitis C virus, pibrentasvir

## Abstract

**Background & Aims:**

Adequate adherence to hepatitis C virus (HCV) treatment is believed to be a key component of treatment success because non‐adherence can potentially result in treatment failure and the emergence of resistant viral variants. This analysis assessed factors associated with non‐adherence to glecaprevir/pibrentasvir (G/P) therapy and the impact of non‐adherence on sustained virological response at post‐treatment week 12 (SVR12) rates in HCV genotype (GT) 1‐6‐infected patients.

**Methods:**

Adherence was calculated by pill counts at study visits during treatment, and defined as having a lowest treatment adherence of ≥80% and ≤120% at each study visit. Exploratory logistic regression modelling assessed predictors of non‐adherence to G/P therapy. SVR12 rates by treatment adherence were assessed in the intent‐to‐treat (ITT) population and modified ITT (mITT) population, which excludes non‐virological failures.

**Results:**

Overall, 97% (2024/2091) of patients were adherent to G/P therapy at all consecutive study visits. Alcohol use was the only baseline characteristic independently associated with non‐adherence to G/P therapy (OR: 2.38; 95% CI: 1.13‐5.01; *P* = .022). In the mITT population, overall SVR12 rates were high both in patients who were adherent to G/P therapy and those who were not (99% [1983/2008] and 95% [58/61] respectively; *P* = .047). Corresponding SVR12 rates in the ITT population were 98% (1983/2024) and 87% (58/67) respectively.

**Conclusions:**

Most patients adhered to G/P therapy. SVR12 rates were high both in patients who were adherent to G/P treatment and those who were not. Patient education on treatment adherence should remain an important part of HCV treatment.

**Clinical trials registration:**

NCT02604017, NCT02640482, NCT02640157, NCT02636595, NCT02642432, NCT02651194, NCT02243293, NCT02446717.

**Abbreviations**
AE: adverse event
DAA: direct‐acting antiviral
G/P: glecaprevir/pibrentasvir
GT: genotype
HCV: hepatitis C virus
ITT: intent‐to‐treat
mITT: modified intent‐to‐treat
OR: odds ratio
OST: opioid substitution therapy
PWID: people who inject drugs
SOF: sofosbuvir
SVR12: sustained virological response at post‐treatment week 12
TEAE: treatment‐emergent adverse event

**Key points**

This pooled analysis of glecaprevir/pibrentasvir (G/P) in HCV GT1‐6‐infected patients across eight phase 3 clinical trials demonstrated high adherence to G/P treatment as well as high SVR12 rates in both adherent and non‐adherent patients.

## 1 INTRODUCTION

In response to the high burden of chronic hepatitis C virus (HCV) disease, the World Health Organization has set a goal of eliminating chronic HCV infection as a major global public health threat by 2030.^1^ Well‐tolerated, simple, short‐duration, pan‐genotypic direct‐acting antiviral (DAA) regimens with high cure rates as measured by sustained virological response at post‐treatment week 12 (SVR12) will play an important role in realizing this goal.^2^, ^3^, ^4^ Adequate adherence to treatment is believed to be a key component of treatment success because non‐adherence can potentially result in treatment failure and the emergence of resistant viral variants.^5^

Previous analyses of DAA regimens have demonstrated high adherence to treatment in the overall chronic HCV‐infected patient population,^6^, ^7^, ^8^ including those who were on opioid substitution therapy (OST) and people who use drugs^9^, ^10^, ^11^, ^12^, ^13^, ^14^ (≥95% and ≥90% respectively). Some studies have suggested that adherence to DAA therapy decreases with increased treatment duration.^7^, ^8^, ^15^, ^16^ In a pooled analysis of 4825 chronic HCV‐infected patients from 13 trials receiving 8‐24 weeks of HCV antiviral therapy, a longer treatment duration was associated with a lower likelihood of adherence (odds ratio [OR]: 0.98 for each additional week; *P* = .002).^8^ Another identified risk factor for poor treatment adherence includes alcohol use. In the PREVAIL study of models of HCV care for people who inject drugs (PWID), alcohol use was a significant predictor (OR: 2.2; 95% confidence interval [CI]: 1.0‐4.8; *P* = .04) of poor adherence (<80%) to HCV treatment.^16^ Age has been identified in some studies as a risk factor for poor treatment adherence, with lower treatment adherence reported in both elderly (>55 years old) and young (<40 years old) patients.^5^ However, a thorough assessment of risk factors for poor treatment adherence has not been performed for ‘next‐generation’ DAA therapies. Moreover, the impact of poor adherence on SVR12 rates has not been evaluated for short‐duration 8‐week DAA regimens. In phase 2 studies of the sofosbuvir [SOF]/velpatasvir/voxilaprevir DAA regimen, ultra‐short treatment durations of 4 or 6 weeks resulted in high rates of virological relapse.^17^, ^18^ Therefore, it is particularly important to determine how ‘forgiving’ 8‐week DAA regimens are when treatment adherence is poor.

Once‐daily, coformulated glecaprevir/pibrentasvir (G/P) 300/120 mg is a next‐generation, pan‐genotypic DAA regimen given for a short, 8‐week duration in treatment‐naive patients without cirrhosis, and a 12‐week duration in treatment‐naive patients with compensated cirrhosis.^19^, ^20^, ^21^ In pooled analyses of HCV genotype (GT) 1‐6‐infected patients in phase 2 and 3 clinical trials, G/P demonstrated overall intent‐to‐treat (ITT) SVR12 rates of 98% (943/965) in patients without cirrhosis treated for 8 weeks^22^ and 96% (297/308) in patients with compensated cirrhosis treated for 12 weeks (treatment duration was 16 weeks for HCV GT3‐infected patients with prior treatment experience with interferon [IFN]/ pegylated IFN, ribavirin and/or SOF [PRS] and HCV GT1‐infected patients with prior treatment experience with a non‐structural [NS] protein 5A and/or NS3/4A protease inhibitor).^23^

This analysis assessed factors associated with non‐adherence to G/P therapy and the impact of non‐adherence on SVR12 rates in HCV GT1‐6‐infected patients who were enrolled in eight phase 3 clinical trials.

## 2 METHODS

### 2.1 Study design

This was a pooled analysis of G/P in HCV GT1‐6‐infected patients with compensated liver disease (with or without cirrhosis) across eight phase 3 clinical trials that have been published previously.^24^, ^25^, ^26^, ^27^, ^28^ In all eight studies, G/P was orally dosed once daily as three 100/40 mg tablets taken with food, for a total dose of 300/120 mg.^24^, ^25^, ^26^, ^27^, ^28^ G/P was administered for 8, 12 or 16 weeks, according to HCV genotype, prior treatment experience and cirrhosis status.^24^, ^25^, ^26^, ^27^, ^28^

All patients provided written informed consent. Studies were conducted in accordance with the International Conference on Harmonization guidelines and the principles of the Declaration of Helsinki. Study protocols were approved by the ethics committees or institutional review boards at each of the participating study sites. All authors had access to study data, reviewed and provided feedback on all versions of the manuscript, and made the decision to submit the manuscript for publication.

### 2.2 Patients

Patients were ≥18 years old, with chronic HCV GT1‐6 infection, without cirrhosis or with compensated cirrhosis. In SURVEYOR‐II Part 4, ENDURANCE‐1, ‐2, ‐3 and ‐4 and EXPEDITION‐1 and ‐4, patients were HCV treatment‐naive or PRS‐experienced.^24^, ^25^, ^26^, ^27^ In MAGELLAN‐1 Part 2, enrolled patients had failed at least one prior NS3/4A protease inhibitor‐ and/or NS5A inhibitor‐containing therapy.^28^ There was no upper limit on patient age or body mass index. A total of 711, 1264 and 116 patients received G/P for 8, 12 and 16 weeks respectively.

### 2.3 Adherence

Patients were allocated study medication at baseline and at week 4 and 8 visits; for those receiving G/P for 16 weeks, patients were also allocated study medication at week 12 visit. Adherence was calculated by dividing the number of pills taken as determined by pill counts at study visits in weeks 4, 8, 12 and 16, and at time of study drug discontinuation (if applicable), by the number of pills expected to be taken. Adherence was evaluated prospectively in all clinical trials. Adequate treatment adherence is commonly defined as taking 80%‐120% of the medication prescribed.^5^, ^29^ Adherence in this study was defined as having a lowest treatment adherence of ≥80% and ≤120% at each study visit. Non‐adherence was defined as having a lowest treatment adherence of <80% or >120% in at least one study visit. For each patient, missing values for drug adherence at any of the treatment visits were imputed with the lowest obtained value from study visits for that patient. Patients with no available adherence data at any treatment visit were considered non‐adherent for this analysis.

### 2.4 Treatment completion

Completion of treatment was defined as treatment durations of ≥52 days, ≥77 days and ≥105 days for 8‐week, 12‐week and 16‐week G/P regimens respectively.

### 2.5 Virological response

SVR12 was defined as HCV < LLOQ (15 IU/mL) 12 weeks after the last dose of study drug without any confirmed quantifiable post‐treatment value through the post‐treatment week 12 visit. On‐treatment virological failure (defined as a confirmed increase >1 log_10_ IU/mL above nadir during treatment, confirmed HCV RNA ≥100 IU/mL after HCV RNA was <15 IU/mL during treatment or HCV RNA ≥15 IU/mL at the end of treatment [with ≥6 weeks of treatment]) and post‐treatment relapse (defined as confirmed HCV RNA ≥15 IU/mL between end of treatment and 12 weeks after the last dose of study drug among patients who both completed treatment and had HCV RNA <15 IU/mL at the end of treatment [excluding reinfection]) were also assessed.^24^, ^25^, ^26^, ^27^, ^28^

### 2.6 Safety

Treatment‐emergent adverse events (TEAEs) were collected from study drug initiation until 30 days after study drug discontinuation. Causality of each adverse event (AE) with respect to study drugs was determined by the study physician. Changes from baseline in laboratory tests and vital sign measurements were also assessed.

### 2.7 Statistical analyses

SVR12 rates by treatment adherence were assessed in the ITT population (defined as all patients who received at least one dose of study drug) and modified ITT (mITT) population, which excludes non‐virological failures. Safety analyses were performed in the ITT population. All CIs were calculated as two‐sided 95% CIs using the Wilson score method for binomial proportions.

Reasons for not achieving an SVR12 in patients who were adherent to G/P therapy were compared with those of patients who were non‐adherent; *P* values were calculated using Fisher's exact test. Descriptive statistics are used to summarize AEs and grade ≥3 laboratory abnormalities.

An exploratory logistic regression medeling assessed predictors of non‐adherence to G/P therapy. Non‐adherence was the dependent variable. Baseline characteristics considered as independent variables were alcohol use (drinker or ex‐drinker versus non‐drinker or unknown); tobacco use (smoker or ex‐smoker versus non‐smoker or unknown); history of depression (yes, no); on stable OST (yes, no); injecting drug use (yes, no); and on polypharmacy (yes [defined as use of ≥5 concomitant medications], no). Baseline alcohol use was determined by asking whether patients had ever used alcohol, with the following categorical answers available: unknown, never, current or former.

## 3 RESULTS

### 3.1 Baseline characteristics

A total of 2091 HCV GT1‐6‐infected patients were included in the pooled analysis of G/P. Overall, 97% (2024/2091) of patients were adherent to G/P therapy at all consecutive study visits. Most (67% [45/67]) patients who were non‐adherent to G/P therapy had a lowest treatment adherence of <80% on at least one study visit; 1% (1/67) of non‐adherent patients had a lowest treatment adherence of >120% on at least one study visit. Adherence data were missing at all study visits for 21 patients; these patients were considered non‐adherent in this analysis. Treatment completion was lower among patients who were non‐adherent to G/P therapy versus those who were adherent (84% [56/67] vs 99% [2003/2024] respectively; *P* < .001). Baseline demographics and disease characteristics for adherent and non‐adherent groups are presented in Table 1. A smaller proportion of patients who were non‐adherent to G/P were aged ≥65 years old versus those were adherent (7% [5/67] vs 14% [288/2024] respectively); however, this difference was not statistically significant (*P* = .116). The adherence rate was 96% (684/711) among those treated for 8 weeks, 97% (1229/1264) among those treated for 12 weeks, and 96% (111/116) among those treated for 16 weeks. The adherence rates by genotype were 98% (920/941) for GT1, 99% (384/388) for GT2, 93% (497/532) for GT3, 97% (150/154) for GT4, 100% (31/31) for GT5, and 92% (34/37) for GT6.

**Table 1:** Baseline demographics and patient characteristics

Characteristic	Adherent^a^ (N = 2024)	Non‐adherent^b^ (N = 67)
Male, n (%)	1107 (55)	43 (64)
Age, median years (range)	54 (19‐88)	49 (20‐69)
Age, ≥65 y, n (%)	288 (14)	5 (7)
Race, n (%)		
White	1603 (79)	56 (84)
Black or African American	124 (6)	4 (6)
Asian	253 (13)	5 (7)
American Indian or Alaska native	12 (0.6)	0
Native Hawaiian or other Pacific Islander	12 (0.6)	2 (3)
Multiple	17 (0.8)	0
Missing	3 (0.1)	0
BMI, median kg/m^2^ (range)	25.8 (17.3‐65.7)	25.1 (18.3‐39.8)
HCV genotype, n (%)		
1	920 (45)	21 (31)
2	384 (19)	4 (6)
3	497 (25)	35 (52)
4	158 (8)	4 (6)
5	31 (2)	0
6	34 (2)	3 (4)
Adherence, n (%)		
<80%	N/A	45 (67)
>120%	N/A	1 (1)
Missing^c^	N/A	21 (31)
HCV RNA, median log_10_ IU/mL (range)	6.1 (0.7‐7.6)	6.3 (1.2‐7.5)
Treatment‐naive, n (%)	1382 (68)	53 (79)
Treatment‐experienced, n (%)		
PRS‐experienced	552 (27)	13 (19)
NS5A and/or NS3A PI‐experienced	90 (4)	1 (1)
Fibrosis stage, n (%)		
F0‐F1	1433 (71)	45 (67)
F2	121 (6)	4 (6)
F3	199 (10)	8 (12)
F4	266 (13)	10 (15)
Missing	5 (0.2)	0
Presence of compensated cirrhosis, n (%)	270 (13)	10 (15)
Severe renal impairment^d^, n (%)	98 (5)	6 (9)
On OST, n (%)	142 (7)	5 (7)
Injecting drug use, n (%)	776 (38)	33 (49)
History of depression, n (%)	411 (20)	16 (24)
On polypharmacy^e^, n (%)	634 (31)	24 (36)
Alcohol use, n (%)		
Drinker or ex‐drinker	1327 (66)	57 (85)
Non‐drinker or unknown	697 (34)	10 (15)
Tobacco use, n (%)		
Smoker or ex‐smoker	1263 (62)	53 (79)
Non‐smoker or unknown	761 (38)	14 (21)
Treatment duration, n (%)		
8 wk	684 (34)	27 (40)
12 wk	1229 (61)	35 (52)
16 wk	111 (5)	5 (7)

Abbreviations: BMI, body mass index; eGFR, estimated glomerular filtration rate; HCV, hepatitis C virus; N/A, not applicable; OST, opioid substitution therapy; PI, protease inhibitor; PRS, prior treatment experience with interferon (IFN) or pegylated (peg) IFN with or without ribavirin (RBV), or sofosbuvir plus RBV with or without pegIFN.

a Adherence was defined as a lowest treatment adherence of ≥80% and ≤120% at each study visit.

b Non‐adherence was defined as a lowest treatment adherence of <80% or >120% in at least one study visit.

c Patients did not have adherence data at any study visit and were considered non‐adherent in this analysis.

d Severe renal impairment was defined as eGFR <30 mL/min/1.73 m^2^.

e Polypharmacy was defined as taking ≥5 concomitant medications.

### 3.2 Baseline predictors of non‐adherence to G/P therapy

The exploratory logistic regression analysis demonstrated that alcohol use was the only baseline characteristic independently associated with non‐adherence to G/P therapy (OR: 2.38; 95% CI: 1.13‐5.01; *P* = .022; Table 2).

**Table 2:** Logistic regression modelling of predictors of non‐adherence to G/P therapy^a^

Baseline characteristic, yes vs no	Odds ratio	95% CI	*P* value
Alcohol use (drinker or ex‐drinker)	2.38	1.13‐5.01	.022
Tobacco use (smoker or ex‐smoker)	1.60	0.82‐3.13	.167
History of depression	0.98	0.53‐1.80	.944
On stable OST	0.81	0.31‐2.11	.660
Injecting drug use	1.06	0.61‐1.84	.830
On polypharmacy (use of ≥5 concomitant medications)	1.10	0.64‐1.87	.737

Abbreviations: CI, confidence interval; G/P, coformulated glecaprevir/pibrentasvir 300/120 mg; OST, opioid substitution therapy.

a Independent baseline variables that were considered in logistic regression modelling were alcohol use (drinker or ex‐drinker vs non‐drinker or unknown); tobacco use (smoker or ex‐smoker vs non‐smoker or unknown); history of depression (yes, no); on stable OST (yes, no); injecting drug use (yes, no); and on polypharmacy (yes [defined as use of ≥5 concomitant medications], no).

### 3.3 Virological response

**Figure 1:**
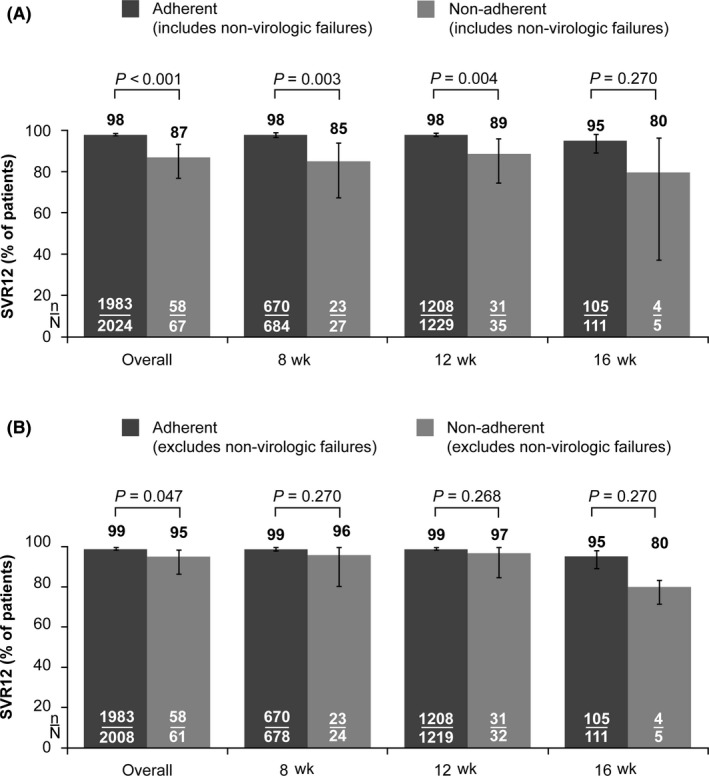
Sustained virological response at post‐treatment week 12 by adherence to G/P therapy and treatment duration in (A) intent‐to‐treat and (B) modified intent‐to‐treat populations Adherent: defined as a lowest treatment adherence of ≥80% and ≤120% at each study visit. Non‐adherent: defined as a lowest treatment adherence of <80% or >120% in at least one study visit. Error bars: two‐sided 95% CIs using the Wilson score method for binomial proportions. Intent‐to‐treat population: defined as all patients who received at least one dose of study drug. Modified intent‐to‐treat population: excludes non‐virological failures. *P* values were calculated using the chi‐squared test (or Fisher's exact test if ≥25% of the cells had expected counts <5) using non‐missing values. Abbreviations: CI, confidence interval; G/P, coformulated glecaprevir/pibrentasvir 300/120 mg; SVR12, sustained virological response at post‐treatment week 12

In the ITT population, SVR12 rates were lower in patients who were non‐adherent versus those who were adherent to G/P therapy (Figure 1A). In the mITT population, which excludes patients with non‐virological failure, there was a marginally significant difference in overall SVR12 rates between patients who were adherent and those who were non‐adherent to G/P therapy (Figure 1B). Although rates of on‐treatment virological failure were low in both patients who were adherent and those who were non‐adherent to G/P therapy, the rate was statistically higher in patients who were non‐adherent (0.3% [7/2024] vs 3% [2/67] respectively [*P* = .03]). Relapse rates were low and similar both in patients who were adherent and those who were non‐adherent to G/P therapy (0.9% [18/1999] vs 2% [1/53] respectively [*P* = .46]). The one case of relapse in a patient who was non‐adherent to G/P therapy was subsequently classified as re‐infection with the same HCV subtype (HCV GT3a) after phylogenetic analysis (clade switch). The rate of premature discontinuation of study drug was higher in patients who were non‐adherent versus those who were adherent to G/P therapy (7% [5/67] vs 0.2% [4/2024] respectively [*P* < .001]). Reasons for failing to achieve SVR12 are given in Table 3. In total, nine patients who were non‐adherent to G/P therapy failed to achieve an SVR12. Most non‐adherent patients (56% [5/9]) failed to achieve SVR12 as a result of premature discontinuation of study drug; three non‐adherent patients (33%) failed to achieve SVR12 as a result of virological failure. Overall, 78% (7/9) were male, 67% (6/9) were <55 years old, 67% (6/9) were infected with HCV GT3, and all nine reported current or past alcohol use. Characteristics of patients who were non‐adherent to G/P therapy and failed to achieve an SVR12 are given in Table 4.

**Table 3:** Treatment outcomes at post‐treatment week 12, by adherence to G/P therapy (intent‐to‐treat population)

Outcome, n (%)	Adherent^a^ (N = 2024)	Non‐adherent^b^ (N = 67)	*P* value
Sustained virological response	1983 (98)	58 (87)	<0.001
Failure to respond	41 (2)	9 (13)	<0.01
Virological failure			
On‐treatment virological failure	7 (0.3)	2 (3)	0.03
Relapse	18^c^ (0.9)	1^d^ (2)	0.46
Failure as a result of other reasons			
Premature discontinuation	4 (0.2)	5 (7)	<0.001
Missing SVR12 data	12 (0.6)	1 (1)	0.35

Note

Intent‐to‐treat population: Defined as all patients who received at least one dose of study drug.

Abbreviations: G/P, coformulated glecaprevir/pibrentasvir 300/120 mg; SVR12, sustained virological response at post‐treatment week 12.

a Adherence was defined as a lowest treatment adherence of ≥80% and ≤120% at each study visit.

b Non‐adherence was defined as a lowest treatment adherence of <80% or >120% in at least one study visit.

c N = 1999.

d N = 53.

**Table 4:** Characteristics of patients who were non‐adherent to G/P therapy and did not achieve SVR12

Reason for non‐SVR12	Assigned treatment duration, weeks	Sex	Age, years	HCV genotype	Alcohol use	Lowest treatment adherence, <80% or >120%	Mean treatment adherence, %	Lowest treatment adherence, %	Timing of lowest treatment adherence, week
Premature discontinuation^a^	12	Male	69	1a	Ex‐drinker	<80	22	22	0‐4
Relapse^b^	12	Male	37	3a	Ex‐drinker	<80	89	71	5‐8
On‐treatment virological failure	8	Male	42	3a	Drinker	<80	N/A	N/A	N/A
Premature discontinuation^c^	12	Male	38	3a	Ex‐drinker	<80	N/A	N/A	N/A
Premature discontinuation^d^	12	Female	46	3a	Ex‐drinker	<80	N/A	N/A	N/A
Missing SVR12 data	8	Female	32	3a	Drinker	<80	N/A	N/A	N/A
On‐treatment virological failure	16	Male	56	3a	Ex‐drinker	<80	91	74	9‐12
Premature discontinuation^e^	8	Male	65	4a	Ex‐drinker	<80	N/A	N/A	N/A
Premature discontinuation^f^	8	Male	30	2b	Ex‐drinker	>120	133	133	0‐4

Abbreviations: G/P, coformulated glecaprevir/pibrentasvir 300/120 mg; N/A, not available; SVR12, sustained virological response at post‐treatment week 12.

a Discontinued on day 27 as a result of adverse event of diarrhoea.

b Subsequently determined to be re‐infection with the same HCV subtype upon phylogenetic analysis (clade switch).

c Discontinued on day 14 as a result of adverse event of diarrhoea.

d Discontinued on day 1 as a result of marital reasons.

e Discontinued on day 36 as a result of non‐compliance.

f Discontinued on day 15 and lost to follow‐up.

### 3.4 Safety

TEAEs and laboratory abnormalities are presented in Table 5. The frequency of TEAEs was numerically higher in patients who were non‐adherent to G/P therapy versus those were who adherent (78% [52/67] vs 67% [1350/2024] respectively); however, the corresponding frequencies of TEAEs that were considered by the study investigator as having a reasonable possibility of being related to study drug were similar (43% [29/67] vs 41% [821/2024] respectively). There were no serious TEAEs related to study drug in patients who were non‐adherent to G/P therapy and one in patients who were adherent (grade 2 transient ischaemic attack, which resolved within 1 day but led to discontinuation of study drug). Rates of study drug‐related TEAEs leading to treatment discontinuation were low in both patients who were adherent to G/P therapy and those who were non‐adherent (0.1% [3/2024] vs 3% [2/67] respectively). There were no deaths among patients who were non‐adherent to G/P therapy and four among patients who were adherent (all deaths were considered by the study investigator as having no reasonable possibility of being related to study drug). Some numerical differences in the frequencies of TEAEs occurring in ≥10% of patients in either group were observed between patient groups; however, the small number of patients who were non‐adherent to G/P therapy should be considered when interpreting the clinical relevance of these findings. Rates of grade ≥3 laboratory abnormalities are presented in Table 5; there were no grade 4 laboratory abnormalities.

**Table 5:** Treatment‐emergent adverse events and laboratory abnormalities by adherence to G/P therapy (intent‐to‐treat population)

	Adherent^a^ (N = 2024)	Non‐adherent^b^ (N = 67)
Safety summary, n (%)		
Any TEAE	1350 (67)	52 (78)
TEAE possibly related to study drug^c^	821 (41)	29 (43)
Serious TEAE	64 (3)	4 (6)
Serious TEAE related to study drug	1 (<0.1)^d^	0
Study drug‐related TEAE leading to discontinuation of study drug	3 (0.1)	2 (3)
Deaths	4 (0.2)^e^	0
TEAEs occurring in ≥10% of patients in any group		
Headache	356 (18)	13 (19)
Fatigue	294 (15)	5 (7)
Nausea	182 (9)	12 (18)
Diarrhoea	115 (6)	8 (12)
Laboratory abnormalities, n (%)		
ALT^f^		
Grade ≥3 (>5 × ULN)	2 (<0.1)	0
AST		
Grade ≥3 (>5 × ULN)	6 (0.3)	0
Total bilirubin		
Grade ≥3 (>3 × ULN)	8 (0.4)	0

Note

Intent‐to‐treat population: Defined as all patients who received at least one dose of study drug.

Abbreviations: ALT, alanine aminotransferase; AST, aspartate aminotransferase; G/P, coformulated glecaprevir/pibrentasvir 300/120 mg; TEAE, treatment‐emergent adverse event; ULN, upper limit of normal.

a Adherence was defined as a lowest treatment adherence of ≥80% and ≤120% at each study visit.

b Non‐adherence was defined as a lowest treatment adherence of <80% or >120% in at least one study visit.

c As assessed by study investigator.

d Grade 2 transient ischaemic attack on day 11 of treatment, which resolved within 1 day without sequelae but led to discontinuation of study drug.

e All deaths were considered by the study investigator as having no reasonable possibility of being related to study drug (adenocarcinoma [n = 1]; cerebral haemorrhage [n = 1]; cause of death unknown pending autopsy [n = 1]; accidental overdose in a patient with a history of opioid overdose [n = 1]).

f Post‐nadir increase; none of the patients with grade 3 ALT elevations had drug‐induced liver injury.

## 4 DISCUSSION

In this analysis of 2091 HCV GT1‐6‐infected patients across eight phase 3 clinical trials, overall adherence to G/P therapy was very high, with only 3% of patients (67/2091) found to be non‐adherent (a lowest treatment adherence of <80% or >120% in at least one study visit). Rates of non‐adherence were low for 8‐, 12‐ and 16‐week treatment durations (3.8% [27/711], 2.8% [35/1264] and 4.3% [5/116] respectively). Treatment completion rates were high in both patients who were non‐adherent to G/P therapy and those who were adherent, although the treatment completion rate was lower in the non‐adherent group. While a smaller proportion of patients who were non‐adherent to G/P were aged ≥65 years old versus those who were adherent (7% [5/67] vs 14% [288/2024] respectively; *P* = .116), this difference was not statistically significant, suggesting that age is not a predictor of adherence to G/P. Alcohol use was the only identified significant independent predictor of non‐adherence to G/P therapy (OR: 2.38; 95% CI: 1.13‐5.01; *P* = .022). This finding is consistent with results from the PREVAIL study of models of care for PWID. In the PREVAIL study, alcohol intoxication was shown to be a significant predictor of non‐adherence to pegylated IFN‐based or all‐oral DAA treatment of chronic HCV GT1 infection (OR: 2.2; 95% CI: 1.0‐4.8; *P* = .04).^16^ Collectively, these results suggest that there may be a group of HCV patients with past or current alcohol use who may benefit from additional support during treatment, such as a daily adherence reminder or psychological counselling.

Neither injecting drug use nor use of OST were predictors of non‐adherence to G/P therapy. These findings are in keeping with a previous analysis of G/P, as well as analyses of other DAA regimens which have shown high rates of adherence (≥90%) in PWID and patients on OST^9^, ^10^, ^11^, ^12^, ^13^; however, recent drug use (within 6 months of study treatment) that, in the opinion of the study investigator, could have precluded adherence to the study protocol was an exclusionary criterion in some of these studies. Patient education on treatment adherence, counselling on harm reduction and peer support should remain an important part of HCV care in the PWID population. Some studies of other DAA regimens have suggested that treatment adherence decreases with treatment duration.^7^, ^8^, ^15^, ^16^ This analysis did not address treatment duration as a predictor of adherence; however, rates of non‐adherence were low regardless of treatment duration.

In the ITT population, there was a slight but significant decrease in SVR12 rates among patients who were non‐adherent versus those who were adherent to G/P therapy (overall SVR12: 87% [58/67] vs 98% [1983/2024] respectively; *P* < .001). This reduction in SVR12 rates was predominately driven by premature discontinuation of study drug and patients lost to follow‐up; there was no pattern in reasons for premature discontinuation (Table 4). Only three in nine non‐adherent patients who failed to achieve an SVR12 did so as a result of virological failure. The virological relapse noted in one of these three patients was subsequently classified as re‐infection with the same HCV subtype after phylogenetic analysis. While rates of on‐treatment virological failure were low in both patients who were adherent and those who were non‐adherent to G/P therapy (0.3% [7/2024] and 3% [2/67] respectively; *P* = .03), the rate of on‐treatment virological failure was statistically higher in those who were non‐adherent to G/P therapy. This finding suggests that non‐adherence to G/P therapy may be associated with an increased risk of on‐treatment virological failure; however, the very small number of non‐adherent patients with on‐treatment virological failure should be considered when interpreting the clinical importance of this finding. In the mITT population, high overall SVR12 rates (≥95%) were achieved in both patients who were adherent and those who were non‐adherent to the G/P regimen. The high SVR12 rates in non‐adherent patients, including those with proposed baseline predictors of non‐adherence, support the potency of G/P and suggest that G/P is a ‘forgiving’ regimen^29^ in patients who are not fully adherent to the G/P regimen.

The small number of patients who were non‐adherent to G/P across clinical trials is an important limitation of this analysis and should be considered when interpreting predictors of non‐adherence and the impact of non‐adherence on SVR12 rates. Twenty‐one patients had no adherence data at any study visit; these patients were considered non‐adherent for the purpose of this analysis, potentially leading to underestimation of treatment adherence. Treatment adherence is commonly measured by pill counts, which can provide empirical evidence of non‐adherence; however, an important limitation of this method is the potential for patients to discard pills before study visits to appear adherent, potentially leading to overestimation of treatment adherence.^29^ Another limitation of pill counts is that it does not provide qualitative data on adherence, for example, dose timing or non‐adherence on sequential days, both of which can have an important impact on treatment outcome.^29^ The results of this study are based on patients in clinical trials who may not be representative of patients in the ‘real world’ or emerging treatment groups such as adolescents or prisoners. However, recent real‐world effectiveness data for G/P have demonstrated very high overall mITT SVR12 rates (≥98%).^30^, ^31^

In summary, this analysis indicates high adherence to G/P treatment as well as high SVR12 rates in those who are not fully adherent to the G/P regimen. While adequate treatment adherence is important to avoid treatment failure and the emergence of resistant viral variants,^5^ the results of this analysis suggest G/P is a ‘forgiving’ regimen^29^ in patients who are not fully adherent to the G/P regimen and that high SVR12 rates can still be achieved. However, as with all medications, patient education on treatment adherence should remain an important part of HCV treatment.

## CONFLICT OF INTEREST

A Brown: Advisor and speaker for, and recipient of research grants from, AbbVie, Bristol‐Meyers Squibb, Janssen, Gilead Sciences and MSD. TM Welzel: Consultant or speaker for AbbVie, Boehringer Ingelheim, Bristol‐Myers Squibb, Gilead Sciences and Janssen. B Conway: Research and grant support from, and participation in advisory boards for, AbbVie, Bristol‐Myers Squibb, Gilead Sciences, Janssen and Merck. F Negro: Grant support from Gilead Sciences; advisor for AbbVie, Gilead Sciences and Merck. N Bräu: Advisor and speaker for, and received grant support from, AbbVie, Bristol‐Myers Squibb, Gilead Sciences and Merck. J Grebely: Consultant/advisor for, or received grant support from, AbbVie, Cepheid, Gilead Sciences, and Merck/MSD. M Puoti: Temporary advisory board and/or speaker at own events for AbbVie, BMS, Boehringer Ingelheim, Janssen, Gilead Sciences, MSD and Roche; research support from Gilead Sciences and MSD. A Aghemo: Grant support from Gilead Sciences and AbbVie; advisory board and speaker for AbbVie, BMS, Gilead Sciences, Janssen and MSD. H Kleine, D Pugatch, FJ Mensa, YJ Chen, Y Lei: current or former employees of AbbVie, Inc; may own AbbVie stock and/or options. E Lawitz: Consultant, advisor and speaker for, or received research/grant support from AbbVie and Gilead Sciences, T Asselah: Clinical investigator, speaker and consultant for AbbVie, Gilead Sciences, Janssen Pharmaceuticals, Merck Sharp & Dohme, and Roche.
